# An extract from *Taxodium distichum* targets hemagglutinin- and neuraminidase-related activities of influenza virus *in vitro*

**DOI:** 10.1038/srep36015

**Published:** 2016-10-31

**Authors:** Chung-Fan Hsieh, Yu-Li Chen, Chwan-Fwu Lin, Jin-Yuan Ho, Chun-Hsun Huang, Cheng-Hsun Chiu, Pei-Wen Hsieh, Jim-Tong Horng

**Affiliations:** 1 Department of Biochemistry and Molecular Biology, College of Medicine, Chang Gung University, Taoyuan, Taiwan; 2 Graduate Institute of Biomedical Sciences, College of Medicine, Chang Gung University, Taoyuan, Taiwan; 3 Department of Cosmetic Science, Chang Gung University of Science and Technology, Taoyuan, Taiwan; 4 Research Center for Industry of Human Ecology, Chang Gung University of Science and Technology, Taoyuan, Taiwan; 5 Molecular Infectious Disease Research Center, Chang Gung Memorial Hospital, Chang Gung University College of Medicine, Taoyuan, Taiwan; 6 Graduate Institute of Natural Products, College of Medicine, Chang Gung University, Taoyuan, Taiwan; 7 Department of Anesthesiology, Chang Gung Memorial Hospital, Taoyuan, Taiwan; 8 Research Center for Emerging Viral Infections, Chang Gung University, Taoyuan, Taiwan

## Abstract

Influenza virus remains an emerging virus and causes pandemics with high levels of fatality. After screening different plant extracts with potential anti-influenza activity, a water extract of *Taxodium distichum* stems (TDSWex) showed excellent activity against influenza viruses. The EC_50_ of TDSWex was 0.051 ± 0.024 mg/mL against influenza virus A/WSN/33. TDSWex had excellent antiviral efficacy against various strains of human influenza A and B viruses, particularly oseltamivir-resistant clinical isolates and a swine-origin influenza strain. We observed that the synthesis of viral RNA and protein were inhibited in the presence of TDSWex. The results of the time-of-addition assay suggested that TDSWex inhibited viral entry and budding. In the hemagglutination inhibition assay, TDSWex inhibited the hemagglutination of red blood cells, implying that the extract targeted hemagglutin-related functions such as viral entry. In the attachment and penetration assay, TDSWex showed antiviral activity with EC_50_s of 0.045 ± 0.026 and 0.012 ± 0.003 mg/mL, respectively. In addition, TDSWex blocked neuraminidase activity. We conclude that TDSWex has bimodal activities against both hemagglutinin and neuraminidase during viral replication.

Influenza virus is an important respiratory pathogen that has caused pandemic outbreaks around the world. Influenza virus outbreaks in recent history include the 2009 swine-origin influenza virus (SOIV, H1N1pdm) pandemic^1^,^2^. In 1997, the avian A/H5N1 virus was found in Hong Kong; this avian influenza virus showed direct bird-to-human transmission and resulted in serious mortality^3^. In 2013, a novel avian influenza virus H7N9 was identified in 12 provinces of China and caused a high mortality rate in humans^4^,^5^.

Influenza virus belongs to the Orthomyxoviridae family of negative RNA viruses and contains eight segments that encode at least 12 viral proteins^6^,^7^. Influenza viruses are classified into three groups, A, B and C, according to their nucleocapsid (NP) and matrix (M) proteins^8^. The influenza virus A infects avian and mammalian species, while the influenza virus B infects humans and seals^9^. Influenza virus C infects humans and pigs^10^,^11^,^12^, although as it is difficult to isolate, there have been few clinical reports of infection^12^. Seasonal epidemics of influenza virus are caused by influenza viruses A and B^13^. Influenza virus A includes different serotypes defined by two surface glycoproteins: hemagglutinin (HA; subtypes 1–18) and neuraminidase (NA; subtypes 1–11)^14^. HA, NA, and matrix protein 2 (M2) are transmembrane proteins on the virus surface^9^. Matrix protein 1 (M1), found under the viral membrane, is a bifunctional protein^15^. The viral proteins NP, polymerase acid protein, polymerase basic protein 1, and polymerase basic protein 2 are components of the viral ribonucleoprotein (vRNP) complex responsible for the replication of viral RNA^9^.

During initiation of virus entry, HA binds sialic acid moieties on cellular membrane receptor glycoproteins^16^. Subsequently, the virus enters the host cells and the virions fuse with the endosomal membrane in the low-pH environment of the endosomes. The vRNPs enter the cell nucleus and commence synthesis of viral RNA. The new viral RNAs are transported into the cytosol for the synthesis of viral proteins. After newly-synthesized vRNPs reaching to the membrane, progeny virions are assembled, and then budded from the cell surface. Before virions are released, the sialic acid is cleaved by NA^9^,^17^. NA function has also been shown to facilitate virus entry to cells, but the mechanism of this is unclear^18^,^19^,^20^.

Two major classes of antiviral drugs, M2 ion channel inhibitors and NA inhibitors, are available to antagonize influenza viruses^21^. The two potent amantadine -derivative M2 ion channel inhibitors, amantadine and rimantadine, are effective against influenza A virus but not influenza B virus^22^,^23^. However, the frequency of adamantine-resistant influenza virus A is increasing^22^. For example, SOIV and avian H5N1 have demonstrated amantadine resistance^2^,^24^, while influenza virus A/H3N2 has almost 100% adamantine resistance^22^,^25^. The two NA inhibitors, oseltamivir and zanamivir, are sialic acid analogues that can suppress NA activity to block the release of progeny virions from host cells^26^. Although these NA inhibitors are effective against most influenza A and B viruses, mutations in NA can generate resistant virus, and the sites of mutation in oseltamivir-resistant virus were identified in the catalytic site^27^,^28^. These resistant viruses could in future cause outbreaks and spread around the world, and therefore development of new anti-influenza agents is urgent and important. We screened traditional Chinese herbs and plants to search for new agents with curative effects against NA-resistant strains, which have the potential to cause outbreaks. A new water extract of *Taxodium distichum* stems (TDSWex) was found to exhibit excellent inhibitory activities against NA-resistant strains. Here, we identify the mode of mechanism of TDSWex’s inhibition of influenza virus using several cell-based assays.

## Results

### Antiviral activity and cytotoxicity of the TDSWex

**Figure 1:**
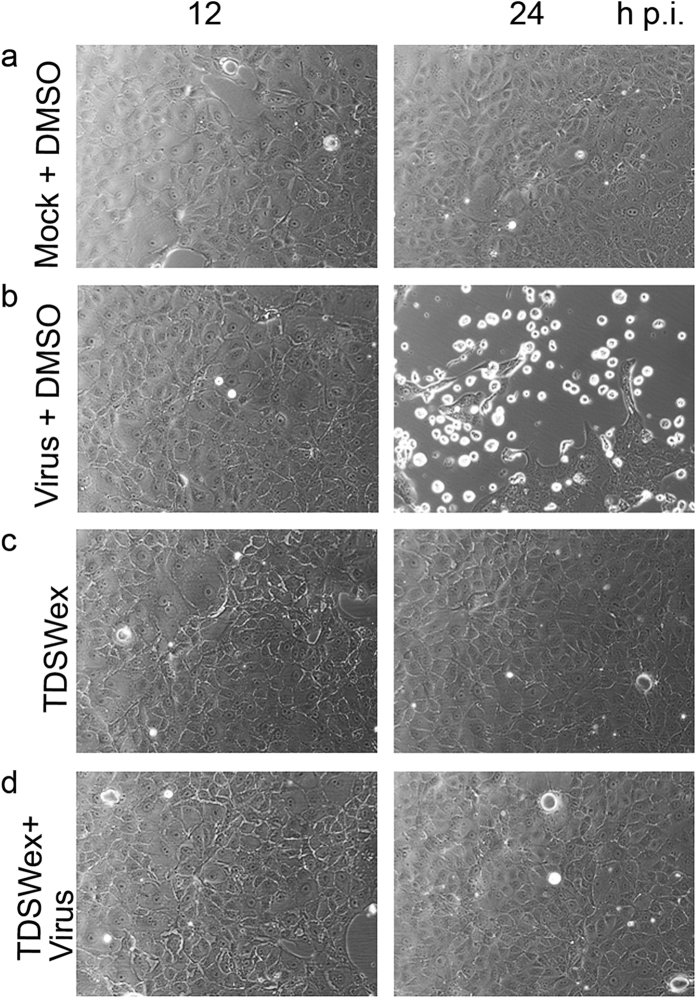
TDSWex inhibited virus-induced CPE in MDCK cells. Cells were infected with WSN (MOI = 0.01) for 1 h and cells were washed twice with HBSS. The infected cells were then incubated with TDSWex (0.1 mg/mL) in E0. The antiviral effect of TDSWex was detected at 12 and 24 h p.i. by microscopy. **(a)** Mock: mock-infection with DMSO (0.05%); **(b)** Virus: cells infected with virus; **(c)** TDSWex: in the presence of TDSWex; **(d)** TDSWex + Virus: cells infected with virus and treated with TDSWex.

First, we screened a panel of Chinese herbs and plants for their antiviral activity, using an assay to measure virus-induced cell death that shows whether a compound has potent antiviral activity. TDSWex revealed an EC_50_ of 0.051 ± 0.024 mg/mL, a CC_50_ of 0.287 ± 0.018 mg/mL, and a selectivity index (SI) of about 5.6 for inhibition of influenza A/WSN/33 virus in Madin–Darby canine kidney (MDCK) cells (Table 1). We demonstrated that the extract can protect cells from virus-induced cell death at a concentration of 0.1 mg/mL (Fig. 1). This concentration of TDSWex did not cause cytotoxicity in MDCK cells within 12, 24, or 72 h (row c of Figs 1 and S1a,b). When challenged by influenza A/WSN/33 virus in the absence of TDSWex, the MDCK cells, as observed under a microscope, appeared more rounded and detached from the dish, an effect defined as cytopathic effect (CPE) (row b, Fig. 1). The extract showed no cytotoxicity and inhibited the virus-induced CPE (rows c and d, Fig. 1). Therefore, we used 0.1 mg/mL TDSWex for the subsequent experiments in this study. This concentration was also below the CC50 for rhabdomyosarcoma (RD) cells (Table 1). TDSWex showed excellent anti-influenza virus activity. It could inhibit not only influenza A/H1N1 virus, but also influenza A/H3N2 virus, SOIV, and recent clinical strains of influenza, including oseltamivir-resistant strains (Table 1). The raw dose/response curve of EC_50_, taking WSN as an example, is shown in Fig. S1c. Three influenza B viruses were tested, and TDSWex again showed anti-influenza virus activity (Table 1). However, it did not exhibit any activity against enteroviruses, adenovirus, or herpes simplex virus 1 (HSV-1), at concentrations up to 1.25 mg/mL (Table 1).

### TDSWex does not suppress viral RNA or protein synthesis when added after viral adsorption

**Figure 2:**
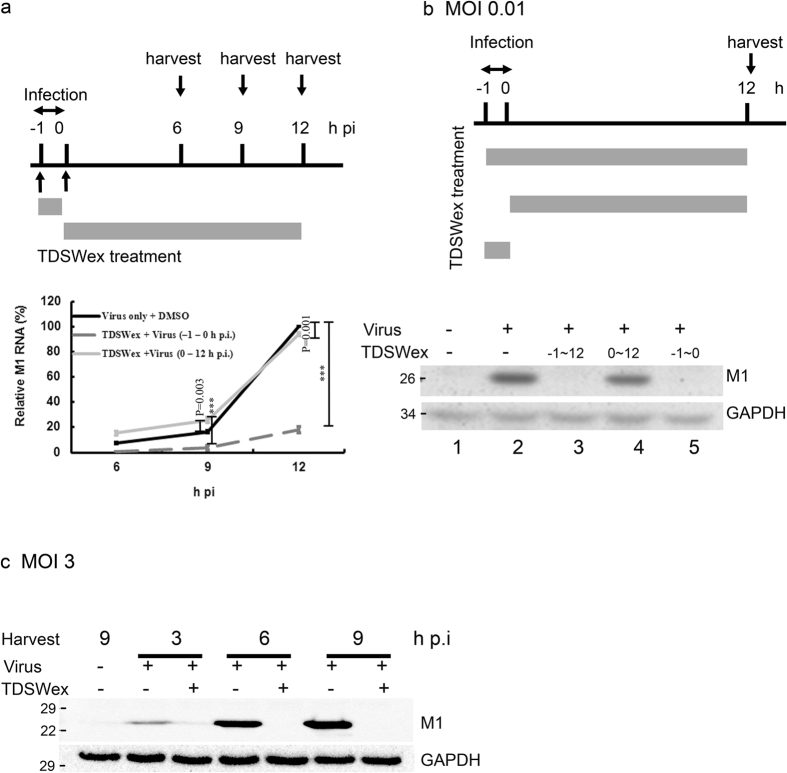
TDSWex treatment in the postadsorption stage could not inhibit viral RNA synthesis and protein production. (**a**) A schematic design of the TDSWex treatment. MDCK cells were infected with WSN at an MOI = 0.01. During or after adsorption, the infected cells were treated with TDSWex (0.1 mg/mL) or DMSO (0.05%), and then harvested at the indicated time p.i. Viral RNA levels were represented by M1 which were normalized by GAPDH through RT-qPCR, and were calculated from three independent experiments. (**b**) A scheme of TDSWex treatment for examining viral protein synthesis. Viral protein expression was represented by M1, with GAPDH as a loading control. This is a representative result of three independent and reproducible experiments. (**c**) Time-of-addition and western blot experiments with an MOI of 3, and collected samples from one single replication cycle. TDSWex was added to the infected cells at –1 h p.i.

**Figure 3:**
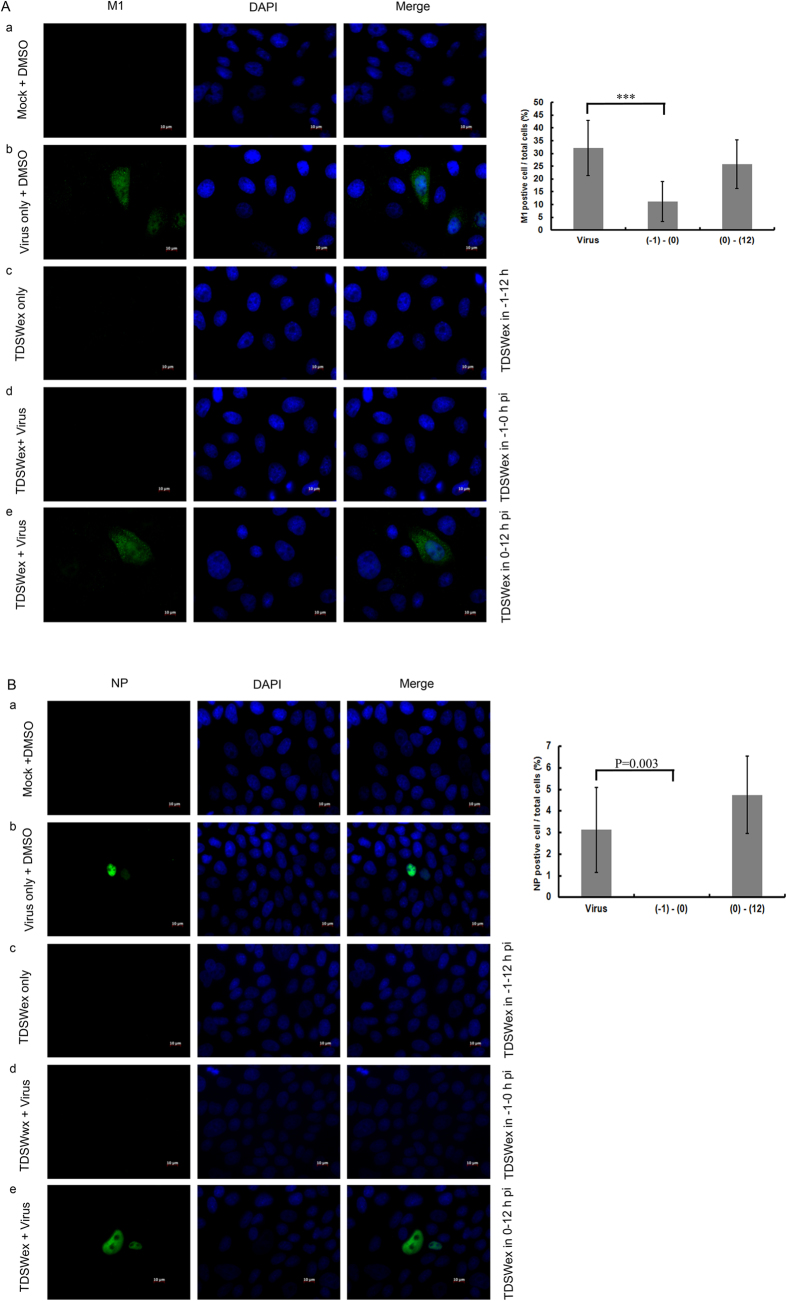
Detection of viral protein expression by immunofluorescence microscopy. MDCK cells were infected with influenza A/WSN/33 (MOI = 0.01). TDSWex (0.1 mg/mL) or DMSO (0.05%) was added to infected cells during or after adsorption and the cells were fixed at 12 h p.i. The green foci represent viral M1 (left column of Fig. 3a) or viral NP (left column of Fig. 3b). The middle column shows nucleus staining with DAPI. The viral protein and DAPI images are merged in right columns. (a) Mock: mock infection with DMSO; (b) Virus only: cells infected with virus; (c) TDSWex only: cells not infected with virus but incubated with TDSWex at –1 to 12 h p.i.; (d) TDSWex + Virus: cells infected with virus and incubated with TDSWex during infection (–1 to 0 h p.i.); (e) TDSWex + Virus: cells were infected with virus and incubated with TDSWex after infection (0 to 12 h p.i.). Right, immunofluorescent foci of M1 and NP of about 300 individual cells from 6 fields were counted in each condition. The ratio of M1- or NP-positive cells to the total cell number was then determined. The data were presented as mean ± SD of the results of two independent experiments. ***p < 0.001 compared to virus only control.

Next, we assessed whether TDSWex blocked viral RNA and protein synthesis (Fig. 2). MDCK cells were infected with influenza A/WSN/33 virus (multiplicity of infection (MOI) = 0.01) for 1 h with or without TDSWex present during viral adsorption. After 1 h, the unbound virus was removed. The horizontal gray bars represent the time during which TDSWex was present. Alternatively, MDCK cells were treated with TDSWex after viral adsorption. The cells were harvested at the indicated time points for analysis using immunoblotting and reverse transcription–quantitative PCR (RT–qPCR) (Fig. 2). Viral RNA synthesis, as indicated by the RNA levels of M1, was suppressed when TDSWex treatment was present during –1 h to 0 h postinfection (p.i.), and the suppression became more noticeable at 12 h p.i. (Fig. 2a). However, viral RNA synthesis was not conspicuously different when MDCK cells were treated with TDSWex after viral adsorption (Fig. 2a). The sharp increase in viral RNA synthesis between 9–12 h p.i. might be a result of the exponential synthesis of viral RNA during the first round of viral infection. The effect of TDSWex on viral protein synthesis, as indicated by the levels of viral protein M1, was detected by western blotting. The production of M1 protein was clearly reduced after treatment with TDSWex during –1 h to 12 h p.i. and –1 h to 0 h p.i. (compare lane 2 with lanes 3 and 5, middle panel of Fig. 2b) but was not changed when TDSWex was added after viral adsorption (compare lane 2 with lane 4, middle panel of Fig. 2b). TDSWex also inhibited the accumulation of viral proteins, as determined by western blot, when inoculated with 3 pfu of virus/cell in a single cycle of viral replication (lower panel of Fig. 2c). This result was confirmed by immunofluorescence microscopy analysis (Fig. 3). In the mock-infected control, no immunofluorescent foci of viral M1 or NP protein were observed, suggesting that the antibodies used are specific (row a, Fig. 3a,b). In the absence of TDSWex, M1 and NP proteins became apparent at 12 h p.i. (row b, Fig. 3a,b). The results were similar, in terms of viral protein synthesis and subcellular distribution, in cells treated with TDSWex during 0–12 h p.i. (row e, Fig. 3a,b). In conclusion, synthesis of M1 and NP proteins was suppressed by TDSWex treatment during –1 h to 0 h p.i. but not during 0 h to 12 h p.i. (rows c and d, Fig. 3a,b). The ratio of M1- or NP-positive cells to the total cell number was significantly reduced by TDSWex treatment during –1 h to 0 h p.i. but not during 0 h to 12 h p.i. (right panels of Fig. 3). Based on these results, we suggest that TDSWex blocks influenza virus replication by inhibition of the viral entry stage (–1 h to 0 h) and does not affect viral transcription, translation, or the subcellular localization of M1 or NP.

### Inhibitory mechanism of TDSWex investigated by a time-of-addition assay

**Figure 4:**
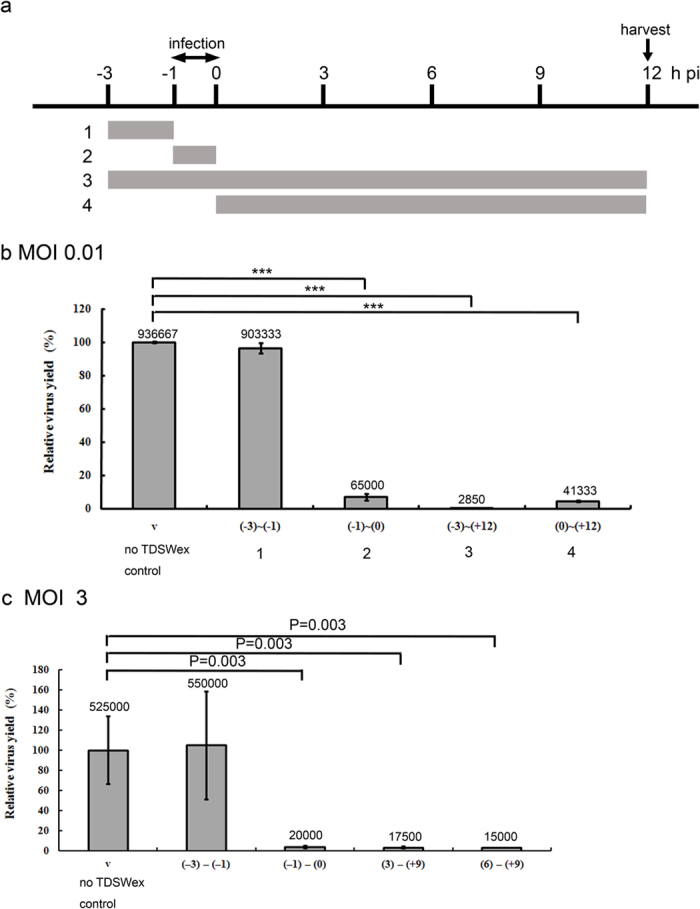
Time-of-addition assay of TDSWex. **(a)** Schematic of the time-of-addition assay. (**b**,**c**) TDSWex (0.1 mg/mL) or DMSO (0.05%) was added at the designated time points. Viral adsorption (MOI of 0.01 or 3) was from –1 h to 0 h p.i. After adsorption, the infected cells were incubated with E0 medium containing TDSWex for the indicated periods. MDCK cells were treated with the extract before infection, from –3 h to –1 h or during infection, from –1 h to 0 h. The culture supernatants were collected at 12 h p.i. and viral yields were determined by plaque assay. The virus yield is presented relative to control ± SD of results from three independent experiments.

To explore the mechanism of the antiviral effect of TDSWex, we used a time-of-addition assay to estimate the virus yields after TDSWex treatment was applied at different stages of viral replication (Fig. 4). The timing of the TDSWex treatment and viral infection is shown schematically in Fig. 4a. First, MDCK cells were preadsorbed with influenza virus during –1 h to 0 h p.i. on ice, and we harvested the culture supernatant at 12 h p.i. for a plaque assay. To assess whether TDSWex affected host cell surface, the extract was added before infection during the period from –3 h to –1 h (Fig. 4b). The viral yields in this sample showed no difference compared with the virus-only control (virus-only control vs. lane 1, Fig. 4b). When TDSWex was present at all stages of the viral replication cycle during the period from –3 h to 12 h p.i., the yields of progeny virus were markedly decreased (virus-only control vs. lane 3, Fig. 4b). When TDSWex was present during the period from –1 h to 0 h p.i. (during adsorption) or from 0 h to 12 h p.i. (after infection), the extract showed excellent protective ability (virus-only control vs. lane 2 and virus-only control vs. lane 4, Fig. 4b). We performed a time-of-addition assay with an MOI of 3. The results showed that TDSWex inhibited different stages of replication, similar to the findings obtained when an MOI of 0.01 was used (Fig. 4c). Based on these data, we suggested that TDSWex might act bimodally on both viral entry and release of progeny.

### The bifunctionality of TDSWex prevents both viral entry and release of progeny

**Figure 5:**
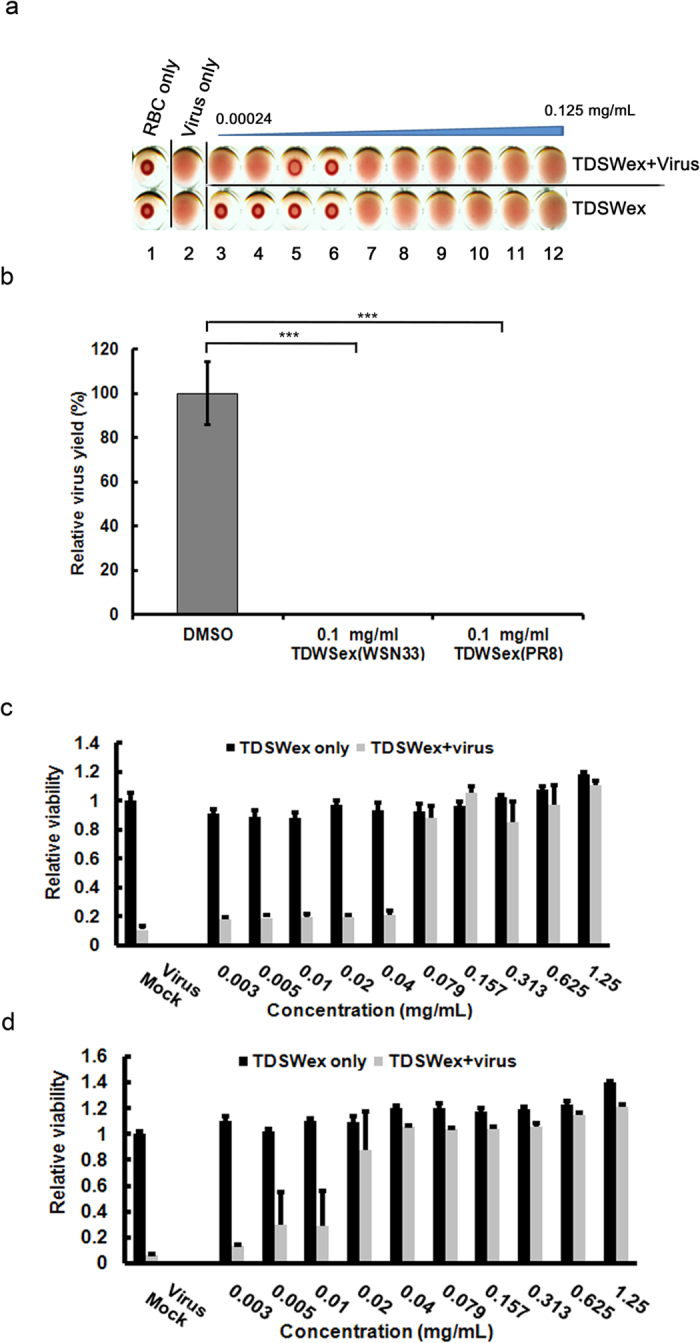
HI, viral particle inactivation, attachment, and penetration assays. **(a)** RBCs were incubated with influenza A/WSN/33 at 4 × HAv in the presence of different concentrations of TDSWex. The controls were RBCs mixed with DMSO (0.06%) in PBS, or virus mixed with DMSO (0.06%) in PBS. Twofold dilutions of TDSWex in PBS from 0.125 mg/mL to 0.00024 mg/mL are shown from right to left. (**b**) Viral particle inactivation assay. The viruses were preincubated with DMSO or TDSWex for 1 h and the resultant mixtures were diluted for titer determination. The viral titer from pretreatment with DMSO was set to 1, and the fold change of viral titers was presented as mean ± SD of the results of two independent experiments. ***p < 0.001. **(c)** Attachment assay. The cells were infected with 3 × TCID_50_ of WSN in the presence of different concentrations of TDSWex on ice for 1 h before incubated at 37 °C for three days. The results are presented as mean viability relative to control ± SD of results from three independent experiments. **(d)** Penetration assay. Cells were precooled before infected with 3 × TCID_50_WSN on ice. After removing unbound virus, the infected cells were incubated with medium containing TDSWex at 37 °C for 1 h followed by acid wash to inactivate the virus on cell surface. The protection against cell death mediated by TDSWex was measured by MTT assay. The results are presented as mean viability relative to control ± SD from three independent experiments.

Before uptake of a virus particle by cellular endocytosis and its passage across the cell membrane, viral envelope protein HA must bind to receptors on host cells^17^,^29^. Therefore, we investigated whether TDSWex targeted the transmembrane viral envelope protein HA using a hemagglutination inhibition (HI) assay to detect the signature effect of HA binding to red blood cells (RBCs). In this assay, twofold serial dilutions of TDSWex were made from 0.125 mg/mL (lane 12, Fig. 5a) to 0.00024 mg/mL (lane 3, Fig. 5a). In the RBC-only control, the RBCs did not agglutinate (lane 1, Fig. 5a). When RBCs were mixed with influenza virus, hemagglutination was detected (lane 2, Fig. 5a). The higher concentrations of TDSWex caused hemolysis (lanes 7–12, lower row, Fig. 5a), but there was no hemolysis at concentrations of TDSWex between lanes 3–6 (lower row, Fig. 5a). Inhibition of hemagglutination was observed at TDSWex concentrations of 0.00195 mg/mL and 0.00098 mg/mL (lanes 5 and 6, upper row, Fig. 5a). This result implies that suppression of viral entry by TDSWex was caused by blockage of the interaction between HA and the sialic acid receptor.

In parallel, the viral particle inactivation assay result showed that both WSN and A/Puerto Rico/8/34 (PR8) strains were significantly inhibited by TDSWex, suggesting that TDSWex might target to and inactivate viral particles (Fig. 5b). We then used biochemical assays to address whether TDSWex blocks viral attachment to and penetration of the host cells in the early stage of viral replication (Fig. 5c,d). In the attachment assay, when MDCK cells on ice were challenged by influenza virus, TDSWex was present in the medium during the adsorption step. The cells were protected from the binding of influenza virus if TDSWex was present, and the EC_50_ was 0.045 ± 0.026 mg/mL (Fig. 5c). In the penetration assay, influenza virus was initially bound to the cellular surface on ice. Unbound virus was removed, followed by endocytosis of the virus at 37 °C in the presence or absence of TDSWex. TDSWex effectively inhibited viral penetration with an EC_50_ of 0.012 ± 0.003 mg/mL (Fig. 5d). These results demonstrate that the mechanism of the antiviral effect of TDSWex is inhibition of the early steps of viral replication during endocytosis, e.g., the disruption of the virus–receptor interaction or of the fusion of the virus with the endosomal membrane.

**Figure 6:**
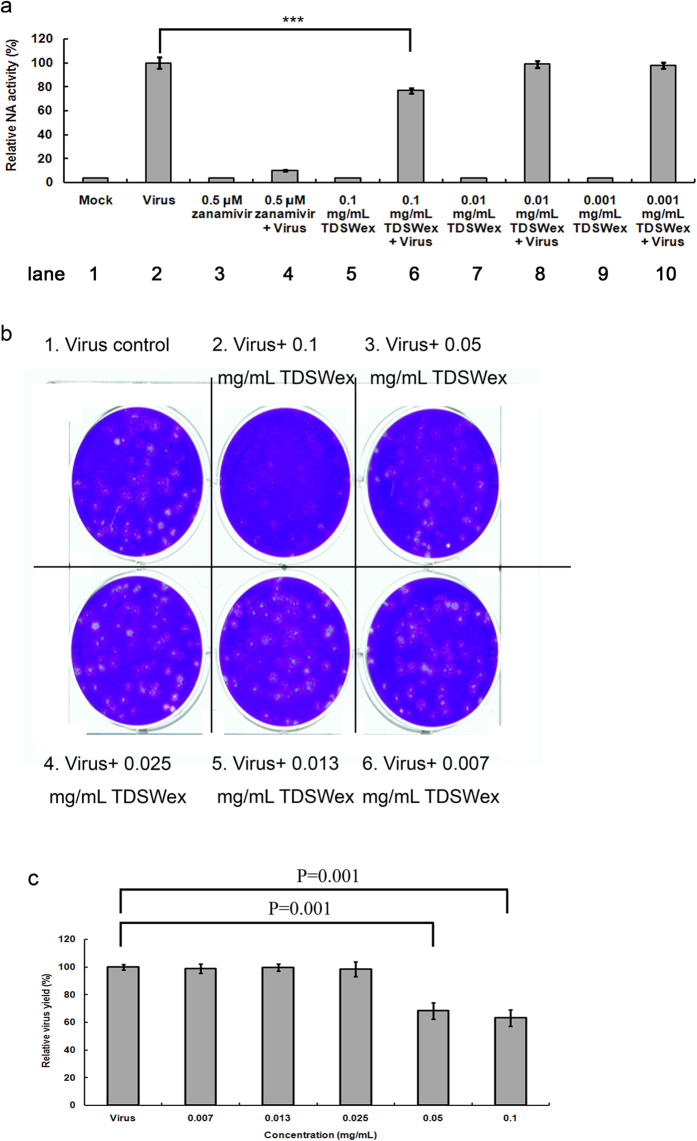
NA assay and plaque reduction assay. **(a)** NA assay. Influenza A/WSN/33 was incubated with TDSWex or zanamivir for 30 min before addition of MU-NANA (60 μM) and then incubated at 37 °C for 1 h. The controls were mock mixed with DMSO (0.3%) or virus mixed with DMSO (0.3%). The results are presented as ratios of relative NA activity ± SD from three independent experiments. **(b)** Plaque reduction assay. MDCK cells were infected with influenza A/WSN/33 and the infected cells were then maintained and overlaid with 0.3% agarose containing the indicated concentrations of TDSWex. The number of plaques was calculated on the right. The data are presented as means ± SD of results from three independent experiments. ***P < 0.001.

To explore whether TDSWex inhibits virus replication through targeting NA activity, we performed NA assay to address this concern. The virus-only control showed NA activity, but the activity was reduced to baseline levels in the mock-treated control and after zanamivir treatment, demonstrating that the enzymatic activity of NA was specific (lanes 1, 2, and 4, Fig. 6a). The NA activity was significantly decreased by about 23% when TDSWex was added at 0.1 mg/mL (lane 6, Fig. 6a). However, lower concentrations of TDSWex (0.01 mg/mL and 0.001 mg/mL, lanes 8 and 10, Fig. 6a) did not inhibit NA activity. Based on these results, we suggest that TDSWex can inhibit NA activity. To confirm whether the antiviral effect of TDSWex correlated with its effect on NA function, we performed a plaque reduction assay^16^,^30^. The plaque numbers were significantly reduced by about 36% and 32%, respectively, when influenza viruses were treated with 0.1 mg/mL or 0.05 mg/mL of TDSWex during viral replication (#1 vs. #2 and #1 vs. #3, Fig. 6b,c). However, no inhibition of virus yield was detected at lower concentrations of TDSWex (#4 to #6, Fig. 6b), indicating that its inhibitory effect on plaque quantity was dose dependent. Overall, we demonstrated that TDSWex functions bimodally against influenza virus by inhibition of both viral entry and NA activity.

## Discussion

Influenza viruses cause heavy morbidity and mortality in pandemics^9^. The emergence of viruses resistant to current therapeutic regimens has raised anxieties about new outbreaks in near future^22^. Therefore, it is crucial to develop new anti-influenza drugs. *T. distichum* is a coniferous tree whose biological activities have been rarely evaluated^31^. *T. distichum* cones showed antifungal and antitermitic activities^32^,^33^, but whether other parts of the plant have antiviral activity is unknown. We screened the extracts of cones, leaves and stems of *T. distichum* for anti-influenza virus activity, and the stem exhibited the best effectiveness against influenza A virus (data not shown). TDSWex was thus purified from the stems of *T. distichum* for our studies.

TDSWex showed inhibitory activity against a broad spectrum of influenza A (including SOIV and resistant strains) and B viruses, but did not show antiviral activity against enteroviruses, adenovirus, or HSV-1, at concentrations up to 1.25 mg/mL (Table 1). A time-of-addition assay was utilized to explore the mechanism of the antiviral effect of TDSWex. The extract did not suppress viral replication when added before virus adsorption (lane 1, Fig. 4b), but we found that it had similar antiviral ability whether cells were treated during or after infection (lanes 2 and 4, Fig. 4b). We further demonstrated that the extract prevented viral binding and NA-related release of viral progeny.

The process of viral replication involves entry, transcription, translation, assembly, and budding^9^. In the time-of-addition assay, TDSWex had inhibitory activity throughout all these stages. First, the extract protected cells against the adsorption of influenza virus (lane 2, Fig. 4b). We used HI, attachment, and penetration assays to confirm that TDSWex inhibited viral entry. Therefore, the reduction in viral RNA and protein synthesis was the result of reduced viral entry (Fig. 2a, lane 5 of Fig. 2b, row d of Fig. 3a,b). In the HI assay, the RBCs did not display influenza virus-induced hemagglutination in the presence of TDSWex (lanes 5 and 6, Fig. 5a). Biochemical assays indicated that TDSWex inhibits both viral adsorption and viral penetration (Fig. 5b–d). We also demonstrated that TDSWex might inactivate both A/WSN and PR8 when applied together in the viral particle inactivation assay, suggesting that the mode of inhibition of TDSWex also applies to other strains of the influenza virus (Fig. 5b). These results imply that TDSWex might disrupt the interaction of HA and the cellular sialic acid receptor, or cause the viral particle to fail to fuse with the endosomal membrane. TDSWex treatment after infection (viral adsorption) also had antiviral activity (lane 4, Fig. 4b). However, because the synthesis of viral RNA and proteins was not significantly affected and there was no difference in their subcellular localization (Fig. 2a, lane 4 of Fig. 2b, row e of Fig. 3a,b), we examined the final stage of viral replication. The release of progeny virions from the host cell surface is dependent on the cleavage of sialic acid by NA^16^. Recent studies demonstrated that NA activity is also involved in viral entry^18^,^19^,^20^,^34^, because if sialic acid is removed, the influenza virus can penetrate host cells^34^. We first demonstrated that TDSWex blocked viral penetration (Fig. 5). In the NA assay, TDSWex treatment reduced the NA activity (lane 6, Fig. 6a). Next, we showed that viral plaque numbers were diminished when cells were treated with TDSWex (Fig. 6b). Based on these results, the extract may restrict both viral entry and release of progeny by inhibition of NA activity. Overall, we suggest that TDSWex possesses bimodal activity against influenza virus for inhibition of viral entry and release of progeny.

There have been reports concerning the chemical composition and biological activities of *T. distichum*. Taxodione and taxodone are two potent antitumor agents^35^,^36^. Shikimic acid is one component of the *T. distichum* extract and is classified as a carbocyclic sialic acid analogue^37^,^38^ that is potentially able to inhibit NA function^38^. Among the variety of compounds that are present in *T. distichum*, quercetin has been reported to inhibit replication of hepatitis C virus, herpesvirus 1, rhinovirus, Japanese encephalitis virus, Mayaro virus, dengue virus type-2 and influenza virus (including H1N1 and H5N1)^39^,^40^,^41^,^42^,^43^,^44^,^45^,^46^. Quercetin exhibited protective efficacy during influenza virus induced-oxidative stress by restoring the concentrations of antioxidants including superoxide dismutase^47^,^48^. In a previous study, quercetin was found to have an inhibitory effect on NA activity^40^. Quercetin also shows significant inhibitory effects on viral translation^49^. However, our results showed that the inhibitory effect of TDSWex was mediated by its ability to block the entry and release of virus, and that it showed little effect on viral protein synthesis. This suggests that the level of quercetin in *T. distichum* may be too low to exert an antiviral effect.

Numerous components including lignans, diterpenes, flavonoids, proanthocyanidins, and sterols were isolated from the genus *Taxodium*^50^,^51^,^52^,^53^. In this study, four lignan stereoisomers (**1**–**4**) were isolated from TDSWex. The backbone structure of compounds **1**–**4** was elucidated as 7,9,9′-trihydroxy-3,3′-dimethoxy-8-*O*-4′-neolignan-4-*O*-*β*-D-glucopyranoside (Fig. S2) by comparing with that reported in previous studies^54^,^55^. The stereochemistry of **1**–**4** at C-7 and C-8 enabled us to assign the structures by using circular dichroism spectroscopy or high-performance liquid chromatography (HPLC) analysis^54^,^55^. Therefore, based on the nuclear magnetic resonance (NMR) data and HPLC profile analyses, the stereo configurations of **1**–**4** were assigned as follows: 7*R*,8*S* (**1**), 7*S*,8*R* (**2**), 7*R*,8*R* (**3**), 7*S*,8*S* (**4**) (Tables S1 and S2, Figs S2 and S3).

In addition, the HPLC fingerprint of TDSWex was established, and compound **4** was identified as the chemical reference with a retention time (rt) of 55.4 min (Fig. S4). The analytic method validation of **4** was carried out, and we obtained a calibration curve with a good linearity ranging from 2.5 to 7.5 μg/mL (Fig. S5). The regression equation and correlation coefficient were y = 92524x – 6410 and *r*^2^ = 0.9993, respectively. The validation data revealed the limit of detection (LOD) to be 1.25 μg/mL, and this was calculated as 13.12 ± 0.89 times the signal to noise ratio (S/N) for the lowest concentration. In addition, the percentage coefficient of variation (%CV) of compound **4** at the LOD was 3.84 while its precision at five different concentrations (7.5, 6.25, 5.0, 3.75, and 2.5 μg/mL) varied between 1.20 and 4.48%. Furthermore, the recovery of compound **4** at 3.0, 5.0, and 7.0 μg/mL was 2.94 ± 0.03, 5.03 ± 0.05, and 6.99 ± 0.03 μg/mL, respectively. These results suggest that the analytic method for compound **4** was reproducible and accurate. Accordingly, the content of compound **4** (2.75 ± 0.03 mg/g, %CV = 2.17) in TDW was determined using HPLC analysis.

## Materials and Methods

### Cell culture and viruses

MDCK cells were cultured in Dulbecco’s modified Eagle’s medium (DMEM; Invitrogen, Carlsbad, CA) with 10% heat-inactivated fetal bovine serum (FBS; JRH Biosciences, Brooklyn, Victoria, Australia), 2 mM l-glutamine (Gibco BRL, Gaithersburg, MD, USA), 0.1 mM nonessential amino acid (NEAA) mixture (Gibco), 100 U/mL penicillin, and 0.1 mg/mL streptomycin (Sigma-Aldrich, St Louis, MO). Human RD cells were maintained in DMEM with 10% heat-inactivated FBS, 100 U/mL penicillin, and 0.1 mg/mL streptomycin. Human lung carcinoma A549 cells were propagated and maintained in minimal essential medium (Invitrogen) supplemented with 10% heat-inactivated FBS and 0.1 mM NEAA. Influenza viruses A/WSN/33 and PR8/34 were obtained from the American Type Culture Collection (ATCC) and were amplified in MDCK cells. Information regarding the sources and the propagation of other influenza viruses, including H1N1pdm, enteroviruses, adenovirus, and HSV-1, was reported previously^56^.

### Preparation of the TDSWex

The stems of T. distichum Rich. were collected from Taipei, Taiwan. The stems (3000 g) were extracted with ethanol (15 L × 2) at 70 °C for 4 h. After evaporation of the solvent in vacuo, the concentrated ethanol extract was partitioned between ethyl acetate and water to give ethyl acetate and water extracts. To prepare the stock of the water-soluble extract of T. distichum for assay, 200 mg of the dry water-soluble extract of T. distichum stem was first dissolved in 1 mL DMSO and incubated at 37 °C in a water bath for 16 h. After sedimentation at 3000 × g for 10 min at 10 °C, the supernatant of the extract was collected and subjected to one more round of centrifugation at 27,000 × g for 30 min. Finally, the aqueous TDSWex was obtained as the supernatant. TDSWex was characterized by HPLC fingerprint and the content of the marker compounds was quantified as described in Supplementary Information. The dried water-soluble extract of T. distichum stem used in the present study was recorded and will be stored for 10 years. The voucher specimen (TDSWex) used in the present study was deposited in the herbarium of Chang Gung University, Taoyuan, Taiwan.

### Cytotoxicity test by 3-(4,5-dimethylthiazol-2-yl)-2,5-diphenyltetrazolium bromide (MTT) assay

Overnight cell culture was incubated with various concentrations of TDSWex in E0 (DMEM containing 100 U/mL penicillin, 0.1 mg/mL streptomycin, 2 mM L-glutamine, 0.1 mM NEAA mixture, and 2.5 μg/mL trypsin) at 37 °C for 12 h or 72 h. The cells were then stained with rinsed MTT as previously described^20^. The 50% cytotoxic concentration (CC_50_) calculated by Reed–Muench method was defined as the concentration of TDSWex that caused death of 50% of the cells.

### Half maximal effective concentration (EC_50_) assay

MDCK cells (2 × 10^4^ cells/well) in a 96-well tissue culture plate were infected with influenza virus A/WSN/33 (9 × TCID_50_ (median tissue culture infective dose), MOI = 2 × 10^–4^) and maintained in different concentrations of the TDSWex in E0. After incubation for 72 h, fixation was performed with 4% paraformaldehyde (PFA) for 1 h at room temperature and the cells were stained with 0.1% crystal violet for 20 min at room temperature. The optical density of the cells was measured with a microplate reader (VICTOR^3^ Multilabel Reader, Perkin Elmer, Shelton, CT). The concentration of TDSWex that could inhibit the virus-induced CPE by 50% calculated by Reed–Muench method was defined as EC_50_.

### CPE observation by microscopy

The MDCK cells in a six-well plate (2.5 × 10^5^ cells/well) were infected with influenza virus at an MOI = 0.01. After adsorption of virus at 37 °C for 1 h, the cells were then washed twice with Dulbecco’s phosphate-buffered saline and 0.1 mg/mL TDSWex in E0 was added. After incubation for the indicated time periods, the CPE was observed with a Zeiss Axiovert 200 M microscope fitted with a 20× objective lens (Carl Zeiss, Göttingen, Germany).

### RNA extraction and RT-qPCR

MDCK cells were infected with influenza virus A/WSN/33 (MOI = 0.01) for 1 h in E0 with or without TDSWex (0.1 mg/mL), and were harvested at indicated time p.i. Total RNA extraction and RT-qPCR were performed as previously described^30^. To quantify changes in gene expression, the ΔCt method was used to calculate the relative changes normalized against GAPDH. Ct is defined as the cycle where fluorescence is determined to be significantly greater than the background. The ratio of vRNA to the internal control was normalized to the 0-h p.i. control level, which was set arbitrarily to 1.0

### Inhibition of viral protein synthesis by TDSWex by western immunoblotting

MDCK cells in six-well plates (5 × 10^5^ cells/well) were infected with indicated MOI of influenza virus A/WSN/33 at 37 °C for 1 h and then washed twice with Hank’s balanced salt solution (HBSS). The cells were treated with or without addition of TDSWex (0.1 mg/mL) in E0 and harvested at the indicated time points for western immunoblotting as previously described^30^. The antibodies against virus M1 antibody (catalogue #1321) were from ViroStat (Portland, ME). The antibody against GAPDH, as an internal control, was from Santa Cruz Biotechnology (SC25778, Santa Cruz, CA). The enhanced chemiluminescence western blotting detection system (Millipore, Billerica, MA) was used for color development.

### Indirect immunofluorescence assay

MDCK cells (1 × 10^5^) on the coverslips were infected or mock-infected with influenza virus A/WSN/33 at MOI = 0.01. After the cells were washed with HBSS, TDSWex (0.1 mg/mL) in E0 was added and the cells were harvested for fixation with 4% PFA for 1 h at room temperature. Permeabilization was done with 0.5% Triton X-100 in phosphate-buffered saline (PBS) for 5 min, followed by blocking with 0.5% bovine serum albumin in PBS for 1 h. The cells were incubation with anti-M1 (catalogue #1321, ViroStat) and anti-NP antibodies (catlogue #ab20343, Abcam, Cambridge, UK) for 1 h. The cell nuclei were stained with 4′,6-diamidino-2-phenylindole (DAPI). Fluorescence of the Alexa-Fluor-488-labeled secondary antibodies and DAPI was detected with a Zeiss Axiovert 200 M microscope with a 100× oil-immersion objective lens.

### Time-of-addition assay

MDCK cells (5 × 10^5^ cells/well) were seeded in six-well plates and then incubated overnight before challenged with influenza virus A/WSN/33 (MOI = 0.01) at –1 h p.i. and kept on ice for 1 h. After viral adsorption, the unbound virus was removed by washing with HBSS. TDSWex (0.1 mg/mL) in E0 was added at –3 to –1 h (preadsorption), –1 to 0 h (adsorption), 0 to 12 h, and –3 to 12 h. All the supernatants were collected at 12 h p.i. for titer determination by plaque assay^30^.

### Viral hemagglutination (HAv) assay and hemagglutination inhibition (HI) assay

Serial twofold diluted influenza A/WSN/33 were made in PBS and mixed with 2× volume guinea pig RBCs in round-bottom 96-well plates for 1 h. The lowest virus titer that caused RBC aggregation was defined as 1 × HAv, and 4 × HAv was used for the HI assay. In the HI assay, different concentrations of TDSWex were incubated with virus on ice for 1 h. This mixture was then incubated with RBCs for 1 h.

### Attachment assay

This assay was previously described with minor modification^30^,^57^. Briefly, MDCK cells were incubated on ice for 20 min before infected with 3 × TCID_50_ of WSN in the presence of different concentrations of TDSWex on ice for 1 h. The medium containing the nonadsorbed virus was removed and the cells were then washed twice with HBSS. The cells were maintained in E0 and cell viability was determined with an MTT assay after incubation for 72 h.

### Penetration assay

This penetration assay was modified from previous reports^30^,^58^,^59^. MDCK cells were chilled on ice for 20 min before infected with 3 × TCID_50_ of influenza virus A/WSN/33 on ice for another 30 min. Different concentrations of TDSWex in E0 were added and the cells were incubated at 37 °C for 1 h. The infected cells were then treated with HBSS (pH 2) for 1 min to inactivate non-penetrated viruses before the addition of HBSS (pH 11) to neutralize the acidic buffer. After removing HBSS, E0 was added and the cells were incubated at 37 °C for 72 h. The effect of TDSWex was determined by the degree of cell viability through MTT assay.

### Viral particle inactivation assay

The WSN and PR8 viruses (5 × 10^5 ^pfu/mL) were incubated with DMSO or 0.1 mg/mL TDSWex for 1 h on ice. The resultant mixtures were diluted up to 30-fold (which was far below the EC_50_) with DMEM for titer determination using a plaque assay.

### NA assay

Influenza virus A/WSN/33 was mixed with different concentrations of TDSWex or zanamivir in sample buffer (32.5 mM MES, 4 mM CaCl_2_, pH 6.5) at 37 °C for 30 min in 96-well tissue culture plates. The substrate, 2′-(4-Methylumbelliferyl)-α-d-N-acetylneuraminic acid (MU-NANA), was added in a final concentration of 60 μM and incubated at 37 °C for 1 h. The reaction was terminated by addition of stop solution (0.1 M glycine buffer containing 25% alcohol, pH 10.7). The fluorescence at an excitation wavelength of 355 nm and an emission wavelength of 460 nm was recorded by Victor^3^.

### Plaque reduction assay

MDCK cells (5 × 10^5^ cells/well) were seeded in six-well plates before infected with influenza virus A/WSN/33 (about 40 plaque-forming units [pfu]) and kept on ice for 1 h. After washing with HBSS, the cells were maintained in indicated concentrations of TDSWex in E0 containing 0.3% agarose. After incubation at 37 °C under 5% CO_2_ for 72 h, the medium was removed and the cells were fixed with 4% PFA for 1 h at room temperature, followed by staining with 0.1% crystal violet for 20 min at room temperature.

### Statistical analysis

All data were presented as the mean or mean ± SEM (standard error of the mean) of three individual experiments. The statistical significance of the difference between two groups was analysed by one-way analysis of variance (ANOVA). A *p* value of <0.05 was considered significant. The actual p value was marked in the figures, unless the *p* value was under 0.001, in which case it was expressed as ***.

## Additional Information

**How to cite this article**: Hsieh, C.-F. *et al*. An extract from *Taxodium distichum* targets hemagglutinin- and neuraminidase-related activities of influenza virus *in vitro*. *Sci. Rep*. **6**, 36015; doi: 10.1038/srep36015 (2016).

**Publisher’s note:** Springer Nature remains neutral with regard to jurisdictional claims in published maps and institutional affiliations.

## Supplementary Material

Supplementary Information

## Figures and Tables

**Table 1:** Inhibition spectrum and cytotoxicity test of TDSWex.

Cell line or virus strain	CC_50_^a^ (mg/mL)	EC_50_^b^ (mg/mL)	SI^e^
Cytotoxic effect			
MDCK^c^	0.287 ± 0.018		
RD	1.162 ± 0.065		
A549	0.397 ± 0.119		
Influenza viruses			
A/WSN/33 (H1N1)		0.051 ± 0.024	5.63
A/PR8/34 (H1N1)		0.017 ± 0.002	16.88
A/TW/5779/98(H1N1)		0.038 ± 0.014	7.55
A/Taiwan/6663/09 (H1N1)^d^		0.006 ± 0.001	47.83
A/Taiwan/7717/09 (H1N1)^d^		0.013 ± 0.002	22.78
A/Taiwan/7855/09 (H1N1)^d^		0.014 ± 0.017	20.50
A/TW/3446/02 (H3N2)		0.041 ± 0.015	7.00
A/TW/70229/03 (H3N2)		0.089 ± 0.034	3.22
A/90167/09 (SOIV)		0.010 ± 0.005	28.7
A/90206/09 (SOIV)		0.020 ± 0.007	14.35
B/TW/70325/05		0.009 ± 0.004	31.89
B/TW/710/05		0.032 ± 0.011	8.97
B/TW/99/07		0.031 ± 0.003	9.26
Enteroviruses			
EV71/Tainan/4643/98		>1.250	
Coxsackievirus A16		>1.250	
Echovirus 9		>1.250	
adenovirus		>1.250	
HSV-1		>1.250	

MDCK or RD cells were infected with influenza virus or enterovirus, respectively, and treated with a serial dilution of TDSWex. The results are mean ± SD of two to three independent experiments.

^a^half the cytotoxic concentration determined by MTT assay.

^b^half the effective concentration determined by inhibition of virus-induced cell death.

^c^EC_50_ for influenza viruses was calculated using MDCK cells and that for enteroviruses was calculated using RD cells. Adenovirus and HSV-1 were tested in A549 cells.

^d^Oseltamivir-resistant strains.

^e^Selectivity Index (SI) is the ratio of CC_50_ to EC_50_.
