# Supplementary material for: An extract from Taxodium distichum targets hemagglutinin- and neuraminidase-related activities of influenza virus in vitro
Source: Sci Rep. 2016 Oct 31;6:36015. doi: 10.1038/srep36015 (PMC5086851; doi:10.1038/srep36015)

An extract from *Taxodium distichum* targets hemagglutinin- and neuraminidase-related activities of influenza virus *in vitro*

Chung-Fan Hsieh[1], Yu-Li Chen[2], Chwan-Fwu Lin[3,4], Jin-Yuan Ho[1], Chun-Hsun Huang[3,4], Cheng-Hsun Chiu[5], Pei-Wen Hsieh[2,4,6,7], and Jim-Tong Horng[1,4,5,8]

[1] Department of Biochemistry and Molecular Biology, College of Medicine, Chang Gung University, Taoyuan, Taiwan

[2] Graduate Institute of Biomedical Sciences, College of Medicine, Chang Gung University, Taoyuan, Taiwan

[3] Department of Cosmetic Science, Chang Gung University of Science and Technology, Taoyuan, Taiwan

[4] Research Center for Industry of Human Ecology, Chang Gung University of Science and Technology, Taoyuan, Taiwan

[5] Molecular Infectious Disease Research Center, Chang Gung Memorial Hospital, Chang Gung University College of Medicine, Taoyuan, Taiwan

[6] Graduate Institute of Natural Products, College of Medicine, Chang Gung University, Taoyuan, Taiwan.

[7] Department of Anesthesiology, Chang Gung Memorial Hospital, Taoyuan, Taiwan.

[8] Research Center for Emerging Viral Infections, Chang Gung University, Taoyuan, Taiwan

Correspondence and requests for materials should be addressed to J.T.H (email: jimtong@mail.cgu.edu.tw)

Materials and Methods

General. The HPLC analysis was performed using a Jasco PU-1580 intelligent HPLC pump, Jasco AS 1555-10 intelligent sampler, and Jassco UV-1575 UV-Vis detector. Preparative HPLC columns (Ascentis® C18, 21.2 mm × 250 mm, and MN, nucleodur C18 THE-C, 10.0 mm × 250 mm) were used for the isolation and separation. An analytical HPLC column (MN, nucleodur C18 THE-C, 4.6 mm × 250 mm) was used to perform the fingerprint chromatography and analytic method validation. The NMR spectra were obtained using a Bruker AVANCE-400 MHz FT-NMR spectrometer with CD_3OD as the solvent.

Extraction and isolation. The TDW (1.2 g) was dissolved in 30 mL of 25% methanol (MeOH) solution and then filtered through a 0.45-μm membrane filter. The filtered TDW solution was separated by using an HPLC preparative column (25% MeOH aq. solution at a flow rate of 5 mL/min) to obtain compounds **1** (4.80 mg, rt 72.9 min), a mixture of **2** and **3** (10.2 mg, rt 75.6 min), and **4** (2.69 mg, rt 82.2 min). Compounds **2** (2.90 mg, rt 59.9 min) and **3** (2.45 mg, rt 67.2 min) were further purified using a preparative column with 11% acetonitrile (MeCN) aq. solution at a flow rate of 2 mL/min.

Establishment of HPLC fingerprint. The TDW solution (2 mg/mL) was dissolved in 20% MeOH aq. solution and filtered before the analysis. The HPLC analysis was

performed by using a Jasco HPLC system with an analytical HPLC column and a mobile phase of 20% MeOH aq. solution at a flow rate of 0.9 mL/min. Moreover, the detector wavelength and injection volume were set at 204 nm and 20 μL, respectively.

Calibration curve and validation. The amount of **4** in the TDW sample was quantified according to a calibration curve constructed at a concentration ranging from 2.5 to 7.5 μg/mL (n = 6). The analytic method was validated by determining the recovery, LOD, and CV. For the recovery test, three concentrations of **4** (3, 5, and 7 μg/mL) were prepared and analyzed. The recovery was determined as the difference between the expected and calculated concentrations from the calibration curve (n = 3). Two concentrations of **4** (0.625 and 1.25 μg/mL) were used for the LOD determination. The S/N was calculated from the area under the concentration-time curve (AUC) and expressed as the mean ± standard error of the mean (SEM). Furthermore, the S/N of the LOD was higher than 10 (n ≥ 6).

Table S1. ^{1}H NMR characteristics of compounds **1-4** (CD_3OD, 400 MHz).

Position	**1**	**2**	**3**	**4**
2	7.09 *d* (1.6)	7.09 *d* (1.6)	7.12 *d* (1.6)	7.12 *d* (1.6)
5	7.11 *d* (8.4)	7.10 *d* (8.4)	7.13 *d* (8.4)	7.13 *d* (8.4)
6	6.94 *dd* (8.4, 1.6)	6.94 *dd* (8.4, 1.6)	6.98 *dd* (8.4, 1.6)	6.98 *dd* (8.4, 1.6)
7	4.86 *d* (5.6)	4.86 *overlap*	4.93 *d* (5.6)	4.93 *d* (5.2)
8	4.29 *m*	4.30 *m*	4.24 *m*	4.23 *m*
2'	6.79 *d* (1.6)	6.79 *d* (1.6)	6.84 *d* (2.0)	6.84 *d* (2.0)
5'	6.82 *d* (8.4)	6.83 *d* (8.0)	6.94 *d* (8.0)	6.94 *d* (8.0)
6'	6.66 *dd* (8.4, 1.6)	6.66 *dd* (8.0, 1.6)	6.70 *dd* (8.0, 2.0)	6.70 *dd* (8.0, 2.0)
7'	2.60 *t* (7.6)	2.60 *t* (7.6)	2.62 *t* (7.6)	2.62 *t* (7.2)
8'	1.79 *m*	1.80 *m*	1.80 *m*	1.80 *m*
9'	3.54 *t* (6.4)	3.54 *t* (6.4)	3.55 *t* (6.4)	3.55 *t* (6.4)
$-OCH_3$	3.78	3.77	3.84	3.84
$-OCH_3$	3.82	3.82	3.84	3.84
1''	4.85 *d* (6.4)	4.86 *overlap*	4.87 *d* (7.6)	4.87 *overlap*

Table S2. Carbon 13 nuclear magnetic resonance (^{13}C NMR) characteristics of compounds **1-4** (CD_3OD, 100 MHz).

Position	**1**	**2**	**3**	**4**
1	138.1	138.1	138.2	138.1
2	112.6	112.7	112.6	112.5
3	150.5	150.4	150.5	150.5
4	147.3	147.3	147.4	147.4
5	117.5	117.4	117.5	117.5
6	121.0	121.0	120.6	120.7
7	73.8	73.8	73.7	73.7
8	86.3	86.4	87.2	87.1
9	62.0	62.1	61.8	61.8
1'	137.7	137.7	137.4	137.3
2'	114.0	114.0	113.9	113.9
3'	151.8	151.8	151.7	151.6
4'	147.1	147.1	147.5	147.4
5'	119.5	119.5	119.3	119.3
6'	121.8	121.8	122.0	122.0
7'	35.5	35.4	35.6	35.6
8'	32.7	32.7	32.7	32.7
9'	62.2	62.2	62.2	62.2
$-OCH_3$	56.5	56.5	56.5	56.5
$-OCH_3$	56.6	56.7	56.7	56.6
1''	102.9	102.9	102.9	102.8
2''	74.9	74.9	74.9	74.9
3''	77.8	77.8	77.8	77.8
4''	71.3	71.3	71.3	71.3
5''	78.2	78.2	78.2	78.2
6''	62.5	62.5	62.5	62.5

Figure S1. Determination of half-maximal cytotoxic concentration (CC_{50}) of TDSWex. MDCK cells were seeded in a 96-well tissue culture plate and treated with concentrations of TDSWex at a two-fold serial dilution from 1.25 to 0.03 mg/mL in E0. Cultures were incubated at 37°C for 12 or 72 h under 5% CO_2 atmosphere. Cells were then washed and stained with MTT.

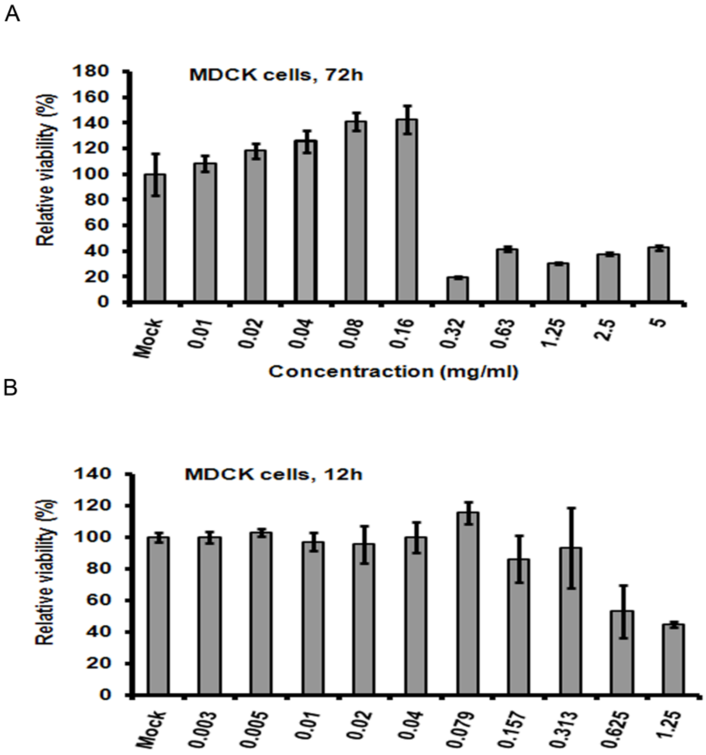

Figure S2. General structure and antiviral activity of four isolated compounds (**1-4**).

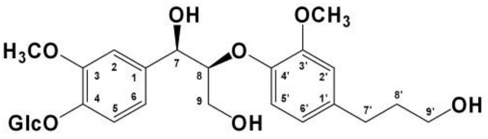

(7*R*,8*S*)-*erythro*-7,9,9′-trihydroxy-3,3′-dimethoxy-8-*O*-4′-neolignan-4-*O*-*β*-D-glucopyranoside (**1**)

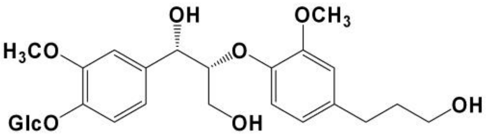

(7*S*,8*R*)-*erythro*-7,9,9′-trihydroxy-3,3′-dimethoxy-8-*O*-4′-neolignan-4-*O*-*β*-D-glucopyranoside (**2**)

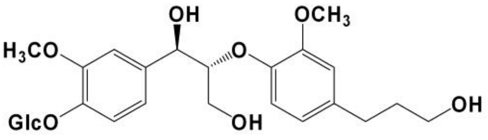

(7*R*,8*R*)-*threo*-7,9,9′-trihydroxy-3,3′-dimethoxy-8-*O*-4′-neolignan-4-*O*-*β*-D-glucopyranoside (**3**)

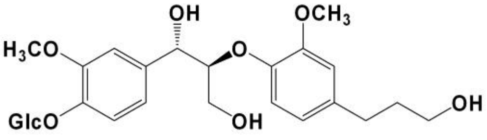

(7*S*,8*S*)-*threo*-7,9,9′-trihydroxy-3,3′-dimethoxy-8-*O*-4′-neolignan-4-*O*-*β*-D-glucopyranoside (**4**)

**Compound**	**EC_{50} (μM)**
**1**	>50
**2**	>50
**3**	>50
**4**	>50

Figure S3. HPLCprofiles of four lignan stereoisomers and TDSWex. (A-D) Compounds **1**, **2**, **3**, and **4** at 10 μg/mL each, with retention time (rt) of 30.7, 31.9, 35.3, and 37.6 min, respectively. (E) Combination of four compounds and (F) TDSWex at 2 mg/mL, respectively. Injection volume, 20 μL; detection wavelength, 204 nm; flow rate, 1.0 mL/min; and mobile phase, 11% acetonitrile (MeCN) aq. solution.

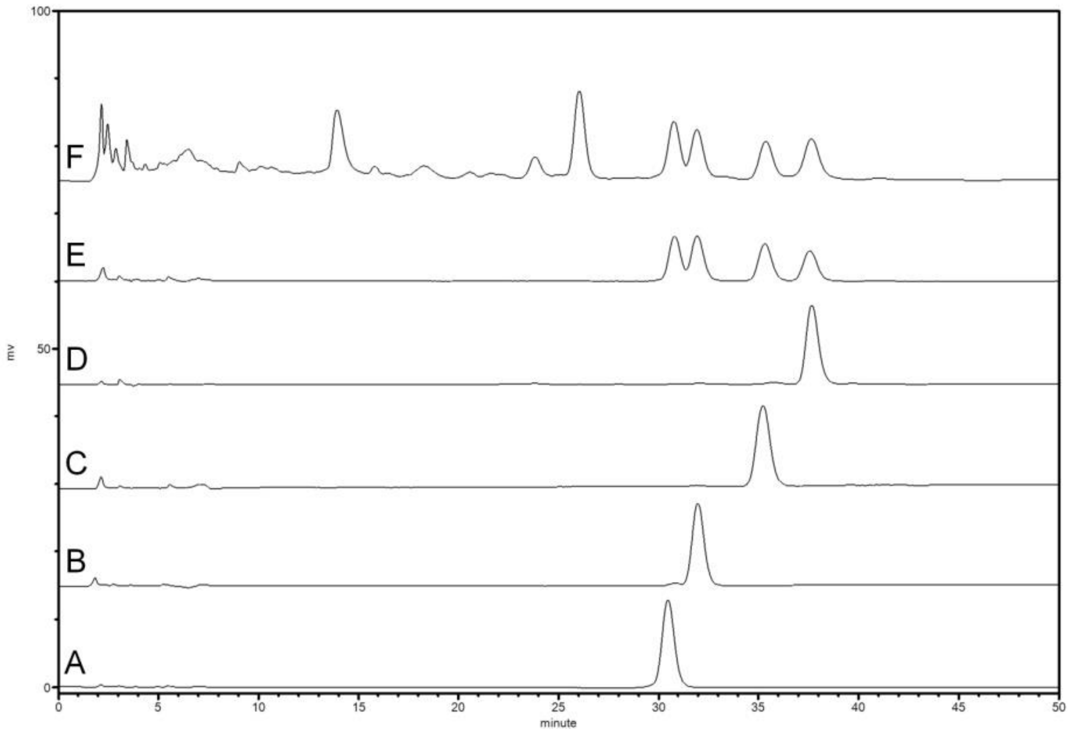

Figure S4. HPLC fingerprint and standard reference of TDSWex. (A) Compound **4**, 5 μg/mL, rt 55.4 min and (B) TDSWex, 2 mg/mL. Injection volume, 20 μL; detection wavelength, 204 nm; flow rate, 0.9 mL/min; and mobile phase, 20% methanol (MeOH) aq. solution.

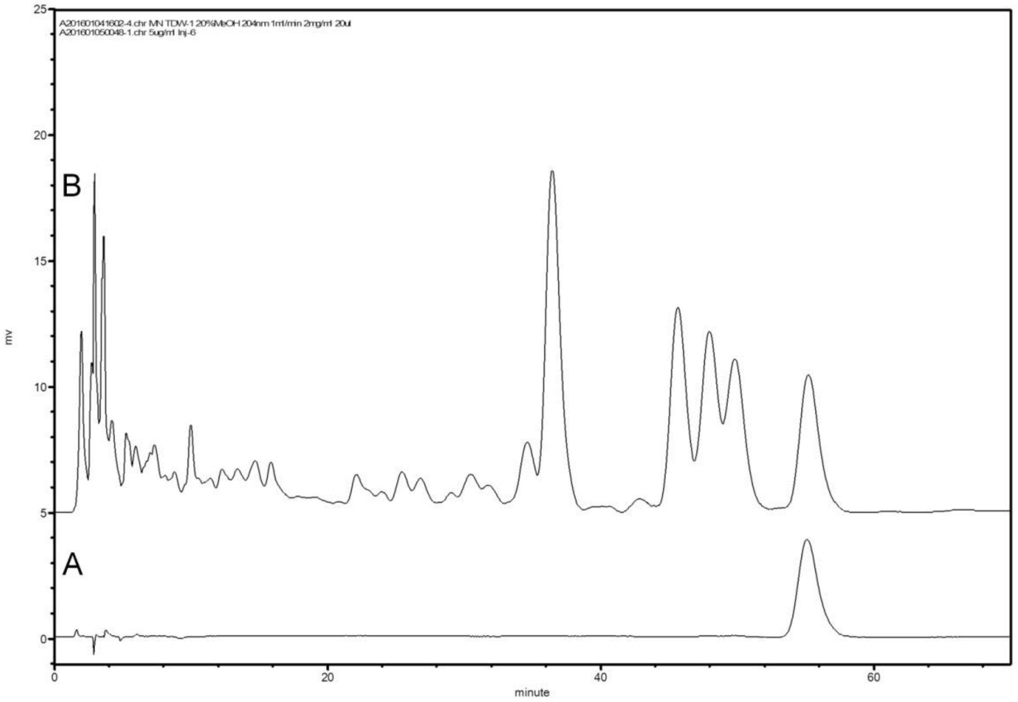

Figure S5. Calibration curve of compound **4** AUC, area under the concentration-time curve.

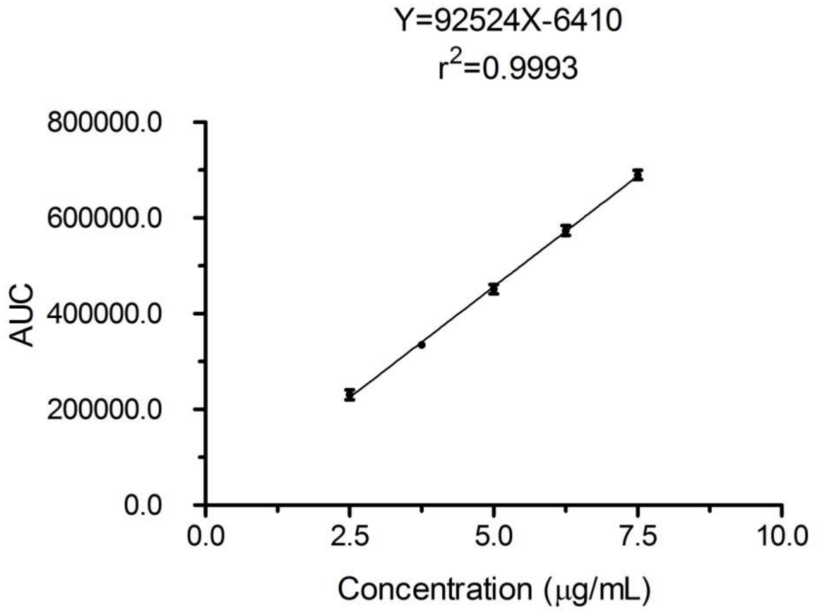

Supplement: Supplementary Information [file srep36015-s1.pdf]
